# MOFGalaxyNet: a social network analysis for predicting guest accessibility in metal–organic frameworks utilizing graph convolutional networks

**DOI:** 10.1186/s13321-023-00764-2

**Published:** 2023-10-11

**Authors:** Mehrdad Jalali, A. D. Dinga Wonanke, Christof Wöll

**Affiliations:** 1 Institute of Functional Interfaces (IFG), Karlsruhe Institute of Technology (KIT), Eggenstein-Leopoldshafen, Germany; 2 Institute of Nanotechnology (INT), Karlsruhe Institute of Technology (KIT), Eggenstein-Leopoldshafen, Germany

**Keywords:** Metal–Organic Frameworks (MOF), Social networking, Machine learning, Materials properties, Guest accessibility, MOFGalaxyNet, Graph convolutional network (GCN)

## Abstract

Metal–organic frameworks (MOFs), are porous crystalline structures comprising of metal ions or clusters intricately linked with organic entities, displaying topological diversity and effortless chemical flexibility. These characteristics render them apt for multifarious applications such as adsorption, separation, sensing, and catalysis. Predominantly, the distinctive properties and prospective utility of MOFs are discerned post-manufacture or extrapolation from theoretically conceived models. For empirical researchers unfamiliar with hypothetical structure development, the meticulous crystal engineering of a high-performance MOF for a targeted application via a bottom-up approach resembles a gamble. For example, the precise pore limiting diameter (PLD), which determines the guest accessibility of any MOF cannot be easily inferred with mere knowledge of the metal ion and organic ligand. This limitation in bottom-up conceptual understanding of specific properties of the resultant MOF may contribute to the cautious industrial-scale adoption of MOFs.

Consequently, in this study, we take a step towards circumventing this limitation by designing a new tool that predicts the guest accessibility—a MOF key performance indicator—of any given MOF from information on only the organic linkers and the metal ions. This new tool relies on clustering different MOFs in a galaxy-like social network, MOFGalaxyNet, combined with a Graphical Convolutional Network (GCN) to predict the guest accessibility of any new entry in the social network. The proposed network and GCN results provide a robust approach for screening MOFs for various host–guest interaction studies.

**Supplementary Information:**

The online version contains supplementary material available at 10.1186/s13321-023-00764-2.

## Introduction

The September 7, 2022, report from the world meteorological organization (WMO) has stipulated that as planet earth continues to warm, wildfires and associated air pollution are expected to increase, which will negatively affect human health and the entire ecosystem. Consequently, the interaction between pollution and climate change will inadvertently impose an additional climate penalty for millions of people across the globe [1]. For this reason, there is a pressing need to design novel tools that can aid with pollution while concurrently striving to reduce emission levels. In this respect, porous materials are most suited for such applications because of their ability to store guest molecules in their pores. However, for a porous material to be used for this purpose, the pore size and channel should be accessible to the guest molecules and chemically tunable to enable selective adsorption of only guest molecules of interest. So far, many porous materials have been investigated and applied for the storage and absorption of chemical compounds. Amongst these materials, metal–organic frameworks, MOFs, are gradually becoming the most promising class of compounds. They are currently amongst the most investigated porous systems because of the facile tunability of their pore sizes and chemistry. Furthermore, their crystallinity greatly simplifies their theoretical description and experimental characterization.

MOFs are porous crystalline materials formed by covalently stitching metal ions or clusters, also referred to as secondary binding units (SBUs) with organic ligands, also known as linkers, in a variety of 2- and 3-dimensional nets or topologies. The unique properties of these porous materials are their low mass densities, high internal surface areas, large pore volumes, facile functionalization, and tuneability of the channels connecting the pores. Consequently, these materials are increasingly being investigated for diverse applications, such as gas storage, filtration, extraction, separation, and catalysis.

To this point, hundreds of thousands of MOFs have been synthesized [2], and millions of hypothetical MOFs have been predicted [3, 4]. This is because of the innumerable ways organic linkers and inorganic building blocks can be combined to produce new MOFs. However, despite the surge in the study and synthesis of MOFs, the industrialization of MOFs has been rather timid. In addition to synthesis scalability, the search for MOFs that fulfil certain requirements for targeted applications is cumbersome due the vast amount of possible MOF structures. Concurrently, we propose that the slow pace of MOF industrialization is in part due to a restricted understanding of the properties of MOFs before they are synthesized. Currently, the properties of MOFs are discerned post-manufacture or predicted using simulations from theoretically conceived models. Consequently, experimentalists with limited computational expertise in simulating the properties of hypothetical MOFs often rely on fate to synthesize a high-performing novel MOF for specific applications. Therefore, there is a yearning need to design a rational bottom-up approach for intelligent crystal engineering of novel MOFs with predefine properties and application before synthesis.

So far, various machine learning (ML), artificial intelligence (AI) techniques, and high-throughput studies have been performed to preselect MOFs with targeted properties before synthesizing them. While it is interesting to note that AI methods have also been used to predict [5] and optimize [6] the synthesis of MOF, ML has frequently been used to considerably accelerate material analysis, where ML models make predictions through learning from a smaller MOF dataset and extend the extracted model to the rest of the materials in the MOF domain. The first ML application in MOFs was implemented to predict the methane storage capacity of MOFs through a support vector machine (SVM) model [7]. In another study, researchers used the random forest and SVM algorithm to train two-class and three-class classification models to predict water stability of MOFs [8]. Snurr’s group also significantly simplified the computational study of MOFs by developing the Quantum MOF database (QMOF) containing approximately 20,000 high-level density functional theory (DFT) geometry-optimized structures of both experimentally synthesized and hypothetical MOFs [9]. In this study, graph neural networks were also implemented to predict the electronic bandgaps of MOFs. In another study, a k-nearest neighbors ML strategy was used to predict the thermal stability of MOFs, which were categorized into four different thermal stability based on the deferential type of MOF descriptors [10].

Although these studies have been used to advance the discovery of MOFs with unique combinations of functionalities, they still suffer from the underlying limitation of working with already established experimental or hypothetical 3D structures of MOFs. However, before synthesis, most experimentalists only have information about the nodes (metal salts) and the organic ligands. Consequently, models that predict the properties of MOFs by using only this information about the constituents as input will be more suitable for a first narrowing down of the MOF chemical spaces containing structures suited for a desired application. In a recent study [11], a new ML model was designed to predict the guest accessibility of MOFs using only information about the linkers and the metal ions. Here, guest accessibility was defined as the diameter of the largest free sphere that a guest molecule can diffuse through the MOF, also known as the pore-limiting diameter (PLD). The study implemented a random forest classifier that predicted the eventual PLD with a remarkable 80.5% certainty. This approach was based on a binary classification that predicts a positive or negative outcome. In this previous study, the PLD was first categorized into four classes, nonporous (PLD < 2.4 Å), small pore (2.4 Å < PLD < 4.5 Å), medium pore (4.5 Å < PLD 8 Å) and large pore (PLD > 8 Å). Then the model predicted a true or a false if the MOF had a PLD that falls into one of the following groups. Inspired by this study, we decided to investigate whether creating a social network of MOFs will outperform the above classifier or, at the very least, be comparable since the network represents a non-binary classification.

Hence in this study, we designed a new approach for predicting the guest accessibility of any MOF by applying a node classification algorithm using graph convolutional networks (GCNs) on a MOF social network, MOFGalaxyNet, created from a social analysis of the metal ions and organic ligands. Social network analysis (SNA) was initially established in the field of social sciences [12, 13] but has been expanded to health informatics [14–17], agriculture [18], life sciences [19, 20], economy [21, 22], and materials science [23, 24]. Graphs are now becoming ubiquitous because of their ability to model complex systems of various kinds, requiring only little adaption to a specific case. In this ML approach, the first step is always to construct a graph containing the elements of a system. Then, the graph can immediately be used to model the relationship between the data object, indicating similarities between MOFs in the current work. Since we aim to predict guest accessibility of MOFs, we take guest accessibility as a label of graph nodes, then define a node classification problem in the graph to predict unlabeled MOFs. Unlabeled MOFs refer to MOFs whose guest accessibility is unknown. In addition, since in real-world applications, there are large amounts of unlabeled data, labeling data is often expensive and time-consuming. The GCN as a node classification method can precisely address this challenge. The GCN is a semi-supervised learning approach over graph-structured data like social networks. It relies on an efficient variant of convolutional neural networks that operates directly on graphs [25]. The GCN can be exploited in many application domains, such as computer vision [26, 27], natural language processing [28, 29], science [30, 31], and others.

## Method

**Fig. 1:**
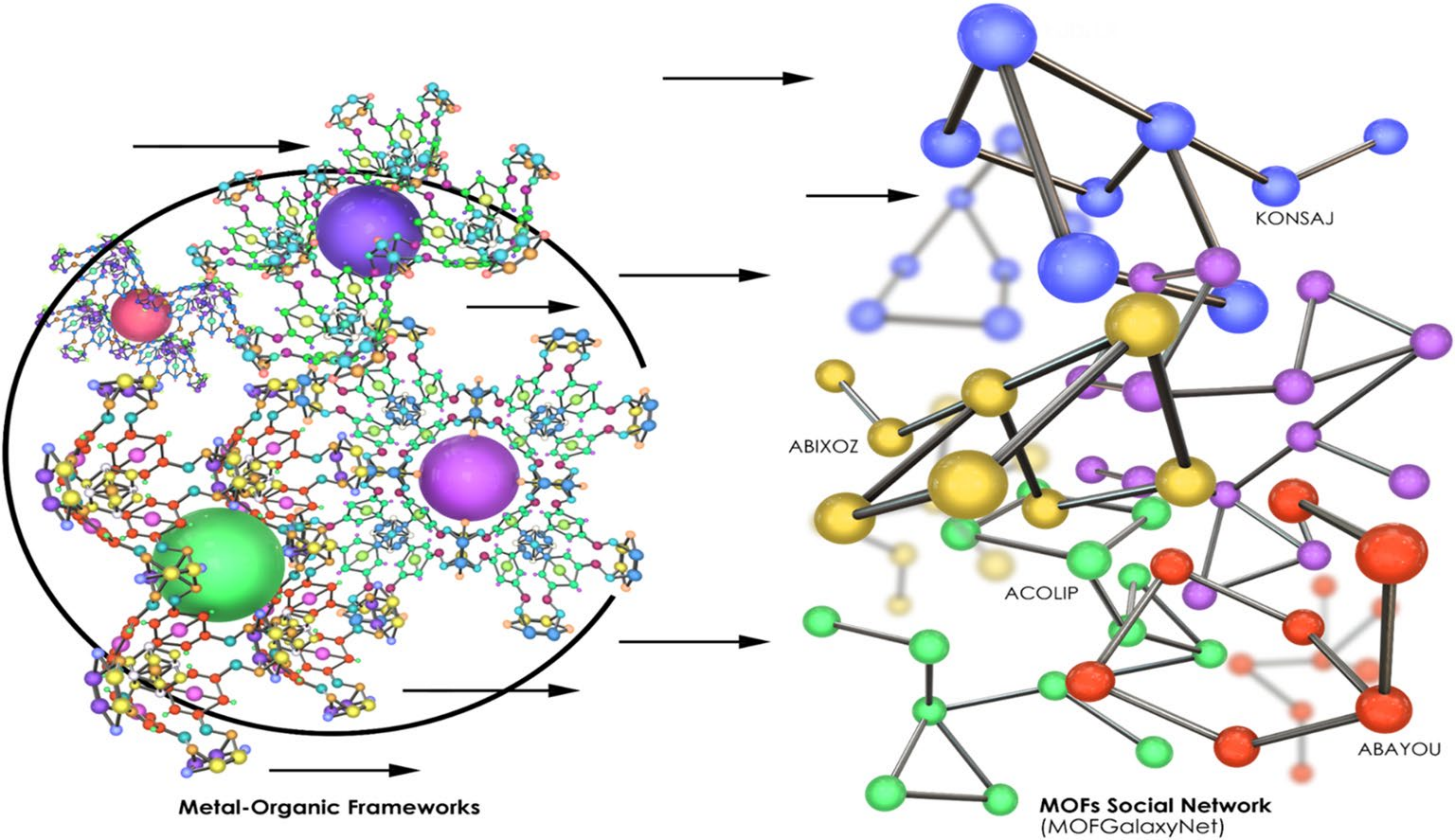
Transforming the metal–organic framework into a social network named as MOFGalaxyNet

In this study, we first utilized social network analysis to create a graph structure employing the data of MOFs. The resulting graph was then analyzed using graph learning methods to make predictions about the properties of new materials. Our primary motive is to develop a new social network platform called MOFGalaxyNet that is built on social network analysis principles and specifically designed to analyze MOFs from their building units—metal ion nodes and MOF linkers (Fig. 1).

**Fig. 2:**
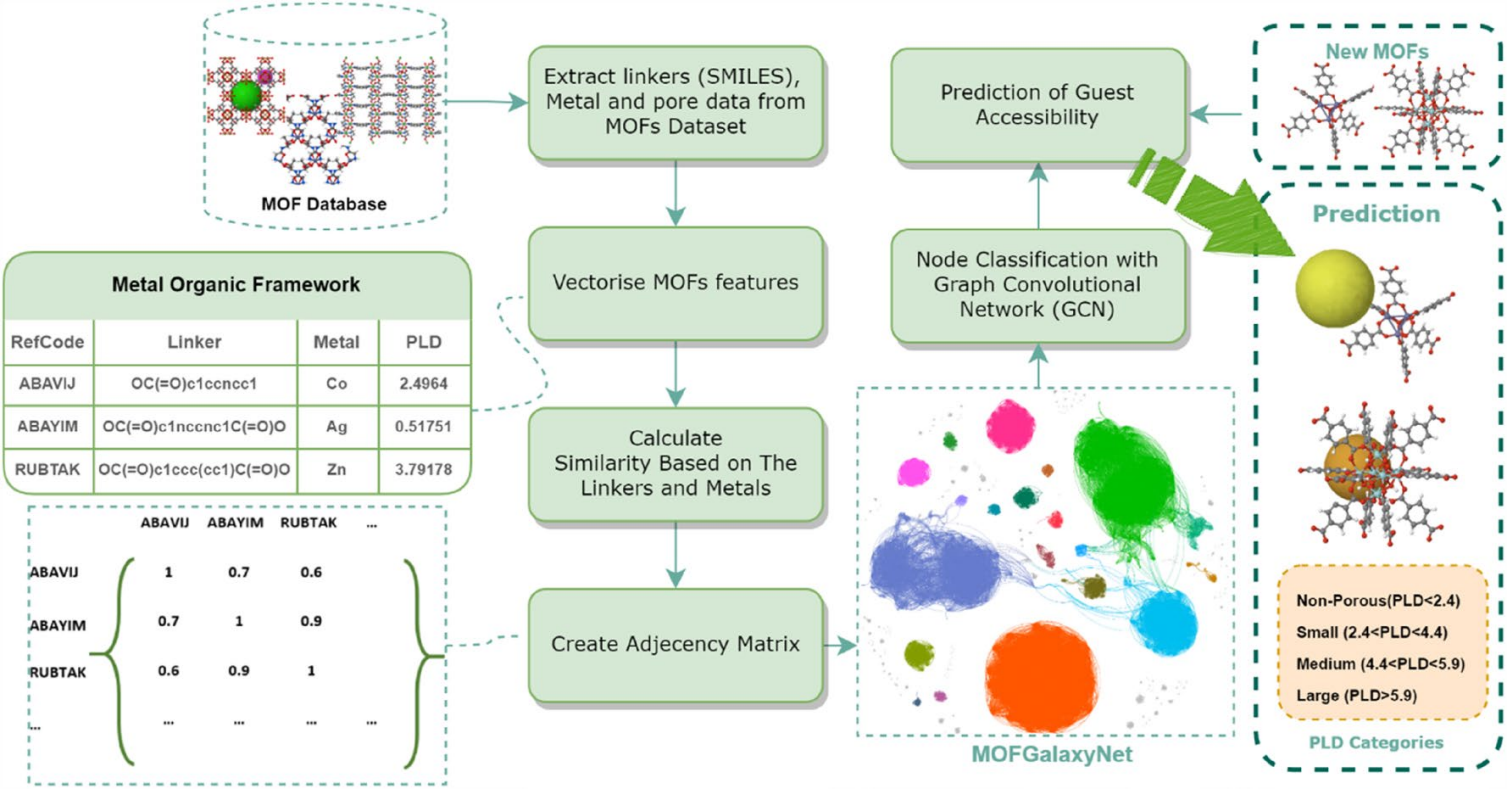
The workflow of constructing MOFGalaxyNet involves utilizing data pertaining to metals and linkers. To build this graph, information from the Metal Organic Framework table is employed, including linker details in SMILES format and metal properties. The PLD column in the table represents the specific property that we aim to predict using MOFGalaxyNet. MOFGalaxyNet functions as a social network that showcases the galaxies of MOFs, providing valuable insights into their characteristics and interactions. Social Network Analysis (SNA) serves as a machine learning technique employed to analyze the graph structure of MOFGalaxyNet. Additionally, the GCN node classification method is utilized to predict guest accessibility by leveraging the information contained within MOFGalaxyNet

MOFGalaxyNet encompasses a vast expanse of Metal–Organic Frameworks (MOFs), forming an extensive collection of interconnected nodes within a weighted and undirected social network. This network representation mirrors the celestial beauty and complexity exhibited by galaxies of MOFs. As the MOF universe expands, MOFGalaxyNet emerges as a collective term to encompass the sheer magnitude of MOFs involved, capturing the massive diversity and interplay among these complex structures. Through MOFGalaxyNet, we explore into uncharted territories, exploring the relationships and interactions within this colossal ensemble, ultimately unraveling new insights, and unlocking the potential of MOFs in various domains. The GCN node classification method was utilized to predict guest accessibility following the construction of MOFGalaxyNet. The entire MOFGalaxyNet workflow is illustrated in Fig. 2.

The key ingredients for constructing the MOFGalaxyNet are the SMILES strings for each organic linker and a set of atomic information for the metal ions. A SMILES (Simplified Molecular Input Line Entry System) string is a concise and easily understandable representation of a molecule’s structure using letters and symbols. It encodes the connectivity and bond types of atoms [32]. These ingredients, SMILES and metal ions, are transformed into vectors and then fed into a module. This module calculates the similarity between different MOFs based on their respective vectors and loads them into an adjacency matrix, which is then used to construct the MOFGalaxyNet. The pore limiting diameter (PLD) can then be predicted using a graph convolutional network (GCN) classification method that is applied to the generated graph. A detailed breakdown of these steps is provided in subsequent sections.

### MOF data preparation

The MOFs used in this study were extracted from a recently published open-access curated MOF database from the Cambridge Structural Database, CSD, that currently contains approximately 12,000 curated structures [33]. To demonstrate the proposed approach, we specifically focused on a subset of 2000 MOFs. This decision was made to ensure that the results and graph visualization are more apparent and comprehensible. By selecting a limited number of MOFs, we aimed to provide a clear and concise demonstration as the first of its kind in this area. The subset of MOFs was chosen from the larger dataset, with the criteria that they were known to consist of only a single organic ligand and a single type of metal ion. This selection allowed us to present a more focused analysis and showcase the effectiveness of the proposed approach.

For this subset of 2.000 MOFs, seven descriptors were extracted for each MOF to build the social network, all represented in Table 1.

**Table 1:** Descriptors of MOFs as properties used in CSD MOFs Dataset. It consists of MOFs’ structure and metal information

MOFs properties	Description
SMILES	String notation for representing the organic ligands present in the MOF
Atomic number	Metal ion information in the form of numerical values
Atomic weight	
Atomic radius	
Mulliken electronegativity	
Polarizability	
Electron affinity	
Pore limiting diameter (PLD)	Porosity information is used to define guest accessibility

We extracted the linker SMILES string notation, metal ion, and pore limiting diameter (PLD) data label from the curated CSD [11]. The metal atomic number, weight, radius, Milliken electronegativity, polarizability, and electron affinity for the metal ion were calculated using the freely available software Mordred [34].

### MOF data vectorization based on the linker and metal information

In machine learning, data vectorization is the process of converting raw data into vectors of real numbers, which can be easily processed by the machine learning algorithms. This is an essential prerequisite because most ML algorithms are designed to be implemented on vectorized data. The dimension of the vector depends on the number of properties used to describe the object. A rectangular vector in ${\mathbb{R}}^{\mathrm{d}}$ can be specified using an ordered set of properties; for instance, an *n*-dimensional vector $\mathrm{\vartheta }$ can be specified using Eq. (1):

1 $$\mathrm{\vartheta }=({v}_{1}, {v}_{2},...,{v}_{n-1},{v})$$

where ${v}_{1}, {v}_{2},...,{v}_{n-1},{v}$ are the components of $\vartheta$.

In this work, it is essential to identify a vector with key properties that are highly correlated with the property of interest of the MOFs. Consequently, our vectorized data is defined by Eq. (2).

2 $$\mathrm{\vartheta }=(\mathrm{SMILES},\mathrm{ AN},\mathrm{AW},\mathrm{AR},\mathrm{ME},\mathrm{P},\mathrm{ EA})$$

where *AN* is the atomic number, *AW* is atomic weight, *AR* is the atomic radius, *ME* is Mulliken electronegativity, *P* is polarizability, and *EA* is electron affinity.

After vectorizing the data, the vectors were normalized. Normalization is necessary to simplify the subsequent analysis by changing the numerical data set on a standard scale without distorting differences in the ranges of values. The most common data normalization method is Min–Max normalization, in which the values are transformed into decimals between 0 and 1, as shown in Eq. (3).

3 $${v}{\prime}=\frac{v-{min}_{A}}{{max}_{A}-{min}_{A}}$$

where ${min}_{A}$ and ${max}_{A}$ denote the minimum and maximum values of the corresponding property A.

### Similarity calculation of MOFs to create an adjacency matrix

Creating an adjacency matrix is the first step in constructing social networks. By definition, a weighted undirected graph can be represented by $g=(\vartheta ,E,A)$ where $\vartheta =\{{v}_{1},{v}_{2},\dots {v}_{N}$} is a set of nodes and ${A}_{N\times N}$ is the adjacency matrix. If there is an edge from ${v}_{i}$ to ${v}_{j}$, then ${A}_{ij}>0$ otherwise ${A}_{ij}=0$ and ${A}_{ij}$ can be defined as edge weight. $\vartheta \in {R}^{N\times d}$ represents the properties of each node. $d$ is the number of feature channels, and $N$ is the number of nodes. ${A}_{ij}$ as the similarity between the MOF vectors can be measured through two methods, one of which is the similarity between SMILES, as mentioned in (4).

4 $${\mathrm{SIM }}_{\mathrm{Linker}}\left({\mathrm{MOF}}_{{\mathrm{A}}_{\mathrm{SMILES}}},{\mathrm{MOF}}_{{\mathrm{B}}_{\mathrm{SMILES}}}\right)=\mathrm{MFS}\_\mathrm{SMILES}({\mathrm{MOF}}_{{\mathrm{A}}_{\mathrm{SMILES}}},{\mathrm{MOF}}_{{\mathrm{B}}_{\mathrm{SMILES}}})$$

where ${MOF}_{{A}_{SMILES}}$ and ${MOF}_{{B}_{SMILES}}$ are two MOFs with corresponding SMILES codes. The similarity between linkers was measured by computing the Morgan fingerprint similarity between the SMILES codes of the MOF.

The Morgan fingerprint is a widely employed technique in the field of cheminformatics, finding applications across diverse domains such as drug discovery, compound similarity analysis, and virtual screening. This method streamlines the comparison of molecular structures and facilitates the quantitative evaluation of their similarities [35]. The Morgan fingerprints analysis involves comparing the Morgan fingerprints of each two MOFs. This comparison utilizes a specific radius parameter for generating Morgan fingerprints. Common bits within the fingerprints represent similar substructures. To determine the similarity between these two MOFs, a similarity coefficient, such as the Tanimoto or Dice coefficient [36], is calculated based on their Morgan fingerprints. The resulting Tanimoto similarity coefficient quantifies the structural resemblance between the MOFs, relying on their Morgan fingerprints. An example can be found in the Additional file 1: Fig S2.

Comparison between the vectors created to encode the metal descriptors can be made using appropriate methods that commonly used in determining vector similarities. Various methods, such as Euclideanm Manhatta, Pearson Product-Moment Correlation coefficient (PPMCC) [37] and the cosine methods, can be used to compare the similarities bewteen vectors. In this study, we used the cosine similarity measure for comparing vectors. This is because this approach is robust and computationally efficient since it involves a simple dot product and vector normalization. In addition, this method is less affected by outliers or noise in the data. The cosine similarity is defined as the cosine of the angle between the vector, see Eq. (5).

5 $${\mathrm{SIM }}_{\mathrm{Metal}}\left({\mathrm{MOF}}_{{\mathrm{A}}_{\mathrm{Metal}}},{\mathrm{MOF}}_{{\mathrm{B}}_{\mathrm{Metal}}}\right)=\mathrm{CosineSimilarity}\left({\mathrm{MOF}}_{{\mathrm{A}}_{\mathrm{Metal}}},{\mathrm{MOF}}_{{\mathrm{B}}_{\mathrm{Metal}}}\right)=\frac{\sum_{\mathrm{i}=1}^{\mathrm{d}}{\mathrm{MOF}}_{{\mathrm{A}}_{{\mathrm{Metal}}_{\mathrm{i}}}}\cdot {\mathrm{MOF}}_{{\mathrm{B}}_{{\mathrm{Metal}}_{\mathrm{i}}}}}{\sqrt{\sum_{\mathrm{i}=1}^{\mathrm{d}}{{\mathrm{MOF}}_{{\mathrm{A}}_{{\mathrm{Metal}}_{\mathrm{i}}}}}^{2}}\cdot \sqrt{\sum_{\mathrm{i}=1}^{\mathrm{d}}{{\mathrm{MOF}}_{{\mathrm{B}}_{{\mathrm{Metal}}_{\mathrm{i}}}}}^{2}}}$$

where ${\mathrm{MOF}}_{{\mathrm{A}}_{\mathrm{Metal}}}$ and ${\mathrm{MOF}}_{{\mathrm{B}}_{\mathrm{Metal}}}$ are d-dimensional vectors of two MOFs. Because the value of linkers and metal similarities might have a different value, the weighted average can be used to equalize the value of similarities to calculate the final similarity as Eq. (6).

6 $$\mathrm{SIM}\left({\mathrm{MOF}}_{\mathrm{A}},{\mathrm{MOF}}_{\mathrm{B}}\right)=\mathrm{\alpha }\times {\mathrm{SIM }}_{\mathrm{Linker}}\left({\mathrm{MOF}}_{{\mathrm{A}}_{\mathrm{SMILES}}},{\mathrm{MOF}}_{{\mathrm{B}}_{\mathrm{SMILES}}}\right)+{(1-\mathrm{\alpha })\times \mathrm{SIM }}_{\mathrm{Metal}}\left({\mathrm{MOF}}_{{\mathrm{A}}_{\mathrm{Metal}}},{\mathrm{MOF}}_{{\mathrm{B}}_{\mathrm{Metal}}}\right)$$

where α is the assigned weight that may be taken from some computational tests. In this work, after testing a range of weights that started from 0.5 (equality of worthiness for both similarities), a weight of 0.7 (α = 0.7) was found to be the most representative. This weight and the threshold value ($\varphi$) explained in the following section directly affect the number of edges and the prediction results. Finally, all the similarities allocated in the adjacency matrix ${A}_{N\times N}$ where $N$ is the number of MOFs, as given in (7).

7 $${\mathrm{A}}_{\mathrm{N}\times \mathrm{N}}=\left[\begin{array}{ccc}\mathrm{SIM}\left({\mathrm{MOF}}_{1},{\mathrm{MOF}}_{1}\right)\mathrm{SIM}\left({\mathrm{MOF}}_{1},{\mathrm{MOF}}_{2}\right)& \cdots & \mathrm{SIM}\left({\mathrm{MOF}}_{1},{\mathrm{MOF}}_{\mathrm{N}}\right)\\ \vdots & \ddots & \vdots \\ \mathrm{SIM}\left({\mathrm{MOF}}_{\mathrm{N}},{\mathrm{MOF}}_{1}\right)\mathrm{ SIM}\left({\mathrm{MOF}}_{\mathrm{N}},{\mathrm{MOF}}_{2}\right)& \cdots & \mathrm{SIM}\left({\mathrm{MOF}}_{\mathrm{N}},{\mathrm{MOF}}_{\mathrm{N}}\right)\end{array}\right]$$

### MOFGalaxyNet graph construction

A graph was constructed from the adjacency matrix described in the previous section. The nonzero values in the matrix represent an edge (link) between two different MOFs with a specific connection weight. Weak edges need to be eliminated to reduce the complexity of the graph, which increases the efficiency of the graph analysis. For this reason all edges whose links were lower than the specified threshold, $\varphi$, were removed. The value of $\varphi$ was selected based on computational test data covering a range of values, some of which are presented in Section. “Results and discussion”. An example of the final edges list is given in Table 2.

**Table 2:** Some edge lists were created based on an adjacency similarity matrix

Source MOFs	Target MOFs	Weight of edges ($\varphi )$
ACENIF	ABAYIO	0.7
ABAYOS	ABAVIJ	0.6
ABAYOU	ACEBOI	0.75
ACENIF	ABAVIJ	0.8

The weight of the edges indicates the similarities between MOFs. Not all edges are significant and can remove those not strong enough

**Fig. 3:**
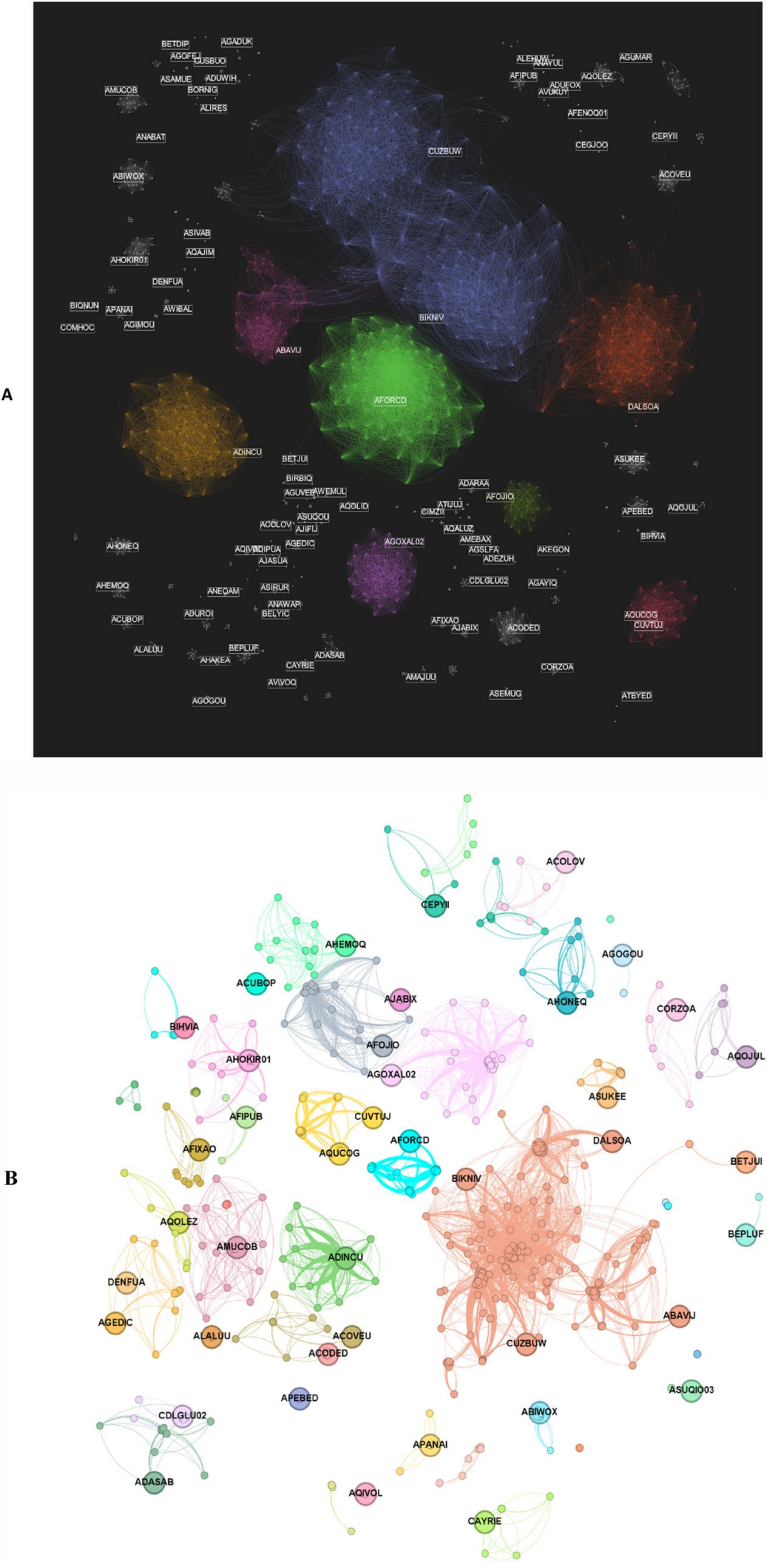
**A** Depicts MOFGalaxyNet as a Galaxy of MOFs employing the MultiGravity Force Atlas technique via Gephi Software. **B** MOFGalaxyNet, consisting of 2000 MOFs, is represented using the OpenOrd layout. To enhance network clarity, a process of sparsification is applied, resulting in the display of only a limited number of MOF labels. The colors of nodes correspond to community nodes determined through the Girvan-Newman method, where nodes of similar colors signify membership in the same community. MOFs with the highest degrees are highlighted in specific communities with their labels. This visualization is created using the OpenOrd layout within the Gephi software

The representation of the sample network of MOFGalaxyNet after eliminating weak edges using a value for $\varphi =0.9$ is shown in Fig. 3. The number of edges decreases to 19266. To improve the clarity of the graph and present a more concise visualization, the graph in Fig. 3. B was consequently sparsified. Sparsification reduces the number of edges in a network while maintaining important topological features [38]. In this study, sparsification was uniquely done to enhance the visual presentation of the graph in order to beautify appearance and enhance readability. It is important to note that this process involves selectively displaying a subset of the graph while maintaining the original structure and integrity of the underlying graph.

**Fig. 4:**
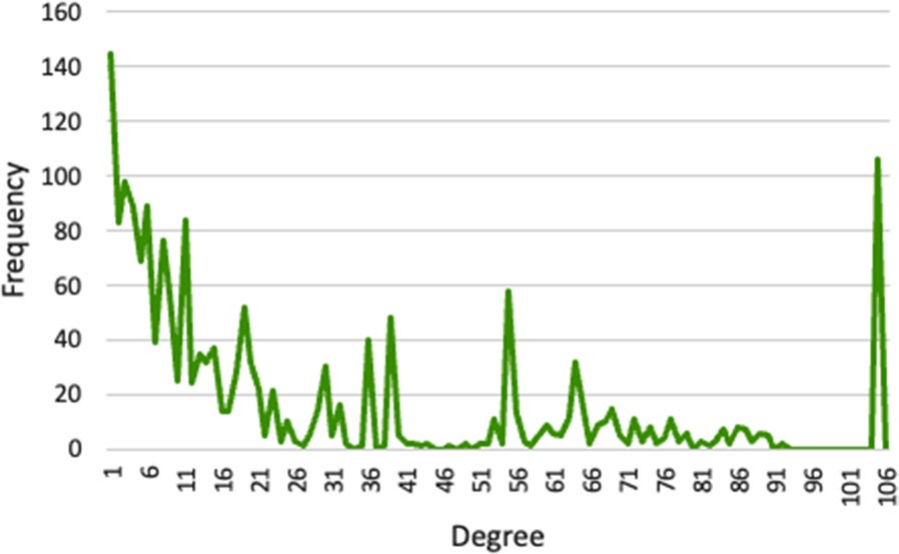
Degree distribution of MOFGalaxyNet—the degree distribution describes the frequency of nodes with a particular degree or number of connections in the network

Following the construction of the social network, we proceeded to examine the resultant MOFGalaxyNet. We started by performing a measure of centrality to assess the relative significance of nodes (or vertices) and links (or edges). In our context, centrality measures the similarity between MOFs within the MOFGalaxyNet. The most straightforward approach is the degree centrality, which for a given node is achieved by counting the number of links connecting it to other nodes. In MOFGalaxyNet, a MOF with a high degree of centrality has similar properties to many other MOFs in the MOFGalaxyNet. The distribution of degree centrality over the whole network is shown in Fig. 4. Moreover, the mean degree within the created MOFGlaxaxyNet is 19.256. Table 3 presents the 15 MOFs with the highest degree in 15 different communities (Cluster) of the MOFs, and illustrates MOFGalaxyNet with these MOFs being highlighted. Additional information pertaining to the network can be found in Additional file 1: Fig S1, S2, as well as Tables S1, S2.

**Table 3:** Ten MOFs with the highest degree in 15 different communities (Cluster) of the MOFs

No	Refcodes	Degree	Community (Cluster-ID)
1	DERBIO	137	0
2	ABIWOX	21	3
3	BELTOD	22	10
4	ACOVEU	17	13
5	ADASEF	15	18
6	ADINCU	54	21
7	AFOJIO	28	32
8	AFORCD	105	33
9	AGOXAL02	39	42
10	AHOKIR01	18	49
11	AMUCOB	16	65
12	BAKGEB	18	79
13	AQUCOG	32	81
14	ASUKEE	18	90
15	BUCWAZ	20	229

We then went further to categorize the graph into subgroups with similar properties through a process of community detection. This was done using the Girvan-Newman algorithm [39] to extract communities effectively. This method is based on the iterative removal of the edges with the highest number of shortest paths between the nodes passing through them. Within MOFGalaxyNet, 246 communities were identified using the Girvan-Newman methodology.

In Fig. 4B, nodes belonging to the same community are represented using the same color. MOFs inside each community are close together. Knowledge of the community is necessary to study the structure of MOFs since investigating certain MOFs represented in each community may enable a detailed understanding of the main properties of other MOFs. 

### Node classification with graph convolutional network

A node classification algorithm on the MOFGalaxyNet was used to predict guest accessibility. This study proposes a more recent node classification algorithm, GCN, based on MOFGalaxyNet. The GCNs are effective techniques for mining knowledge of graph-structured data [5]. As a significant application of graph mining, node classification is applied to several practical domains, such as biomedical, bioinformatics, chemistry, natural language processing, recommendation systems, and other sciences. In addition, many variations of GCN have achieved extraordinary results on these tasks and constantly set up new state-of-the-art performances. The newly proposed GCN performs significantly better on graph-related tasks, such as node classification and recommendation.

**Fig. 5:**
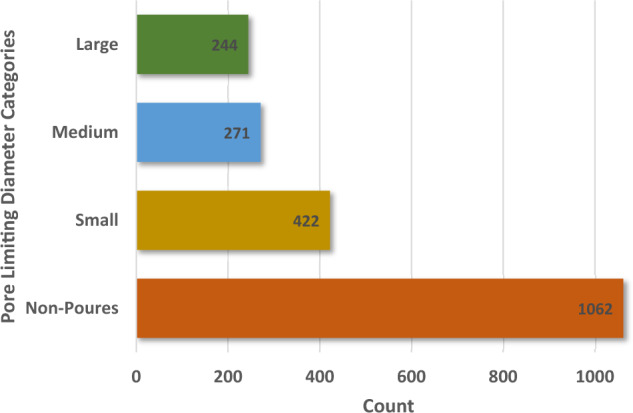
A Histogram of the four categories indicates the selected dataset’s diversity

**Fig. 6:**
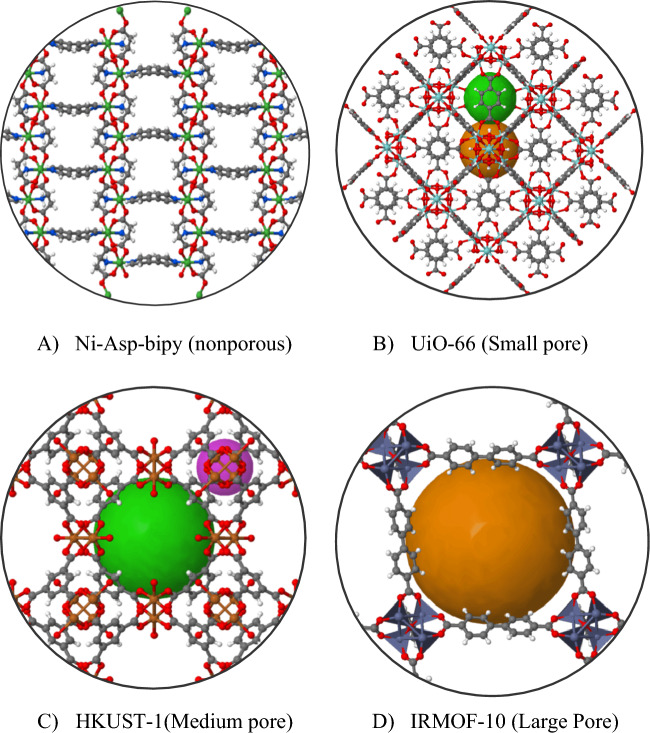
Four examples of MOFs with different PLD sizes. **A** Ni-Asp-bipy is a non-porous MOF, **B** UiO-66 (refcode: RUBTAK) has a PLD of 3.99 Å, making it a MOF with small pores, **C** HKUST-1 (refcode: FIQCEN) has a PLD of 5.23 Å, categorizing it as a MOF with medium-sized pores, **D** IRMOF-10 (refcode: LIHFAK) has a PLD of 12.07725 Å and is considered a MOF with large pores.

The GCN is a convolutional neural network that operates directly on graphs, exploits their structural information, and classifies nodes. First, we considered the node classification problem in MOFGalaxyNet, where the labels defined as PLD are only available for a subset of MOFs. Therefore, the continuous value of PLD must be adapted to four ranges defined in the previous study [11]. The four ranges are defined as nonporous (PLD < 2.4 Å), small pores (2.4 Å < PLD < 4.4 Å), medium pores (4.4 Å < PLD < 5.9 Å), and large pores (5.9 Å < PLD). The histogram of the four PLD categories indicates diversity of the selected MOFs dataset in Fig. 5. For instance, the PLD for IRMOF-10 (refcode: LIHFAK) is 12.07725 Å as large pores MOFs, PLD for HKUST-1 (refcode: FIQCEN) is 5.23 Å as medium pores MOFs, PLD for UiO-66 (refcode: RUBTAK) is 3.99 Å as small pores, and Ni-Asp-bipy is nonporous MOFs (Fig. 6).

**Fig. 7:**
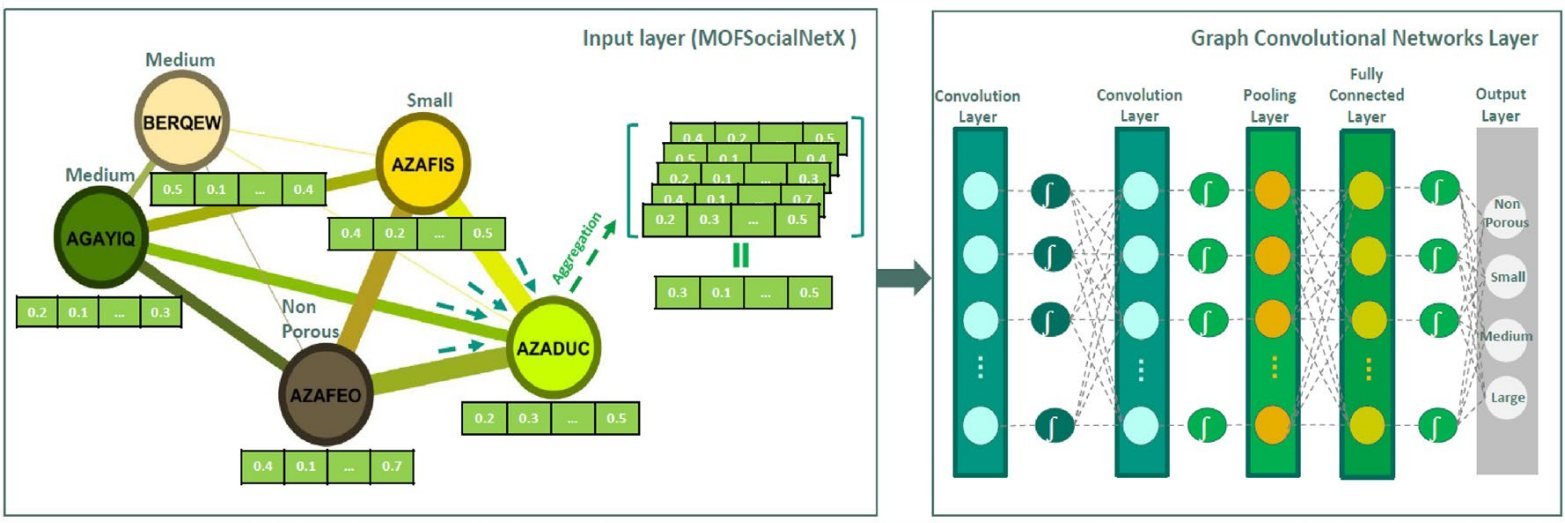
To demonstrate the use of MOFGalaxyNet and GCN node classification for predicting guest accessibility, consider an example. The edges of the graph are depicted with different colors and thicknesses, which correspond to the connection weights. Suppose we want to predict the PLD size for the AZADUC node. To do this, we obtain the feature values of its four neighbors, including AZADUC itself, and apply an aggregation function by taking their average. The resulting value is then fed into the GCN, which returns a PLD size as the predicted label for AZADUC

As a general idea of GCN, for MOFs of each node, we obtained the properties and information of all its neighbors. This information is then quantified using an aggregation function, such as average (i.e., arithmetic mean), as follows:

8 $$\overrightarrow{{\mathrm{MOF}}_{\mathrm{x}}}=\mathrm{Aggregate }\left(\overrightarrow{{\mathrm{MOF}}_{\mathrm{i}}}\right) \,0\le \mathrm{i}\le \mathrm{n}$$

where $n$ is the number of neighbors of $\left\{MOFx\right\}$. The aggregated values are then fed in a convolutional network. In Fig. 7, we provide a simple example using MOFGalaxyNet. Five nodes represent one MOF with the related vector, while the edge with different colors and thicknesses represents the connection weights. We now discuss a specific case, i.e. the prediction of the PLD size for the AZADUC MOF. First, all the feature values of four neighbor nodes are obtained, as well as for the AZADUC node itself are computed and then averaged. The result will be passed through a GCN to return a PLD as the predicted label of AZADUC.

To create a GCN model, a function of MOF properties on MOFGalaxyNet graph $g=(\vartheta ,E,A)$ defined, which takes two main entries, a feature description ${\vartheta }_{i}$ for every node$i$, which is summarized in a $N\times D$ feature matrix $X$, where $N$ is the number of nodes and $D$ is the number of input properties, which is all linker and metal information. Also, a representative description of the graph structure in matrix form will be defined as an adjacency matrix $A$ defined in Section. “Similarity calculation of MOFs to create an adjacency matrix”.

The GCN is a convolutional neural network, and each layer can then be written as a non-linear function as represented in Eq. (9).

9 $${\mathrm{H}}^{(\mathrm{l}+1)}=\mathrm{f}\left({\mathrm{H}}^{\left(\mathrm{l}\right)},\mathrm{A}\right)$$

where ${H}^{(0)}=X$ and ${H}^{(l)}=Z$, $L$ is the number of layers. Generally, it can be defined in a simple form as a layer-wise propagation rule:

10 $$\mathrm{f}\left({\mathrm{H}}^{\left(\mathrm{l}+1\right)},\mathrm{A}\right)=\upsigma \left({\mathrm{AH}}^{\left(\mathrm{l}\right)}{\mathrm{W}}^{\left(\mathrm{l}\right)}\right)$$

where ${W}^{\left(l\right)}$ is a weight matrix for the ${l}^{th}$ convolutional layer, and $\sigma (\cdot )$ is a non-linear activation function like $ReLU$. Despite its simplicity, this model is already rather powerful. In Eq. 10, ${AH}^{\left(l\right)}$ means multiplying $A$ by the hidden layer ${H}^{\left(l\right)}$ for every node. All the feature vectors should sum up all neighboring nodes but not the node itself unless there are self-loops in the graph. If the identity matrix ${I}_{n}$ is added to $A$, it is possible to have self-loops in the graph to consider the node itself for aggregation. Given that $A$ is generally not normalized, multiplication by $A$ will completely change the scale of feature vectors. Normalizing $A$ such that the sum of all rows will be 1, i.e. ${D}^{-1}A$, where $D$ is the diagonal node degree matrix. Multiplying by ${D}^{-1}A$ now corresponds to taking the average of neighboring node properties. In practice, dynamics get more interesting when using a symmetric normalization, i.e. ${D}^{-\frac{1}{2}}A{D}^{-\frac{1}{2}}$ as it does not correspond to a simple average of the neighboring nodes. Therefore, we consider a multi-layer GCN with the following layer-wise propagation rule:

11 $$\mathrm{f}\left({\mathrm{H}}^{\left(\mathrm{l}+1\right)},\mathrm{A}\right)=\upsigma \left({\widehat{\mathrm{D}}}^{-\frac{1}{2}}\widehat{\mathrm{A }}{\widehat{\mathrm{D}}}^{-\frac{1}{2}}{\mathrm{H}}^{\left(\mathrm{l}\right)}{\mathrm{W}}^{\left(\mathrm{l}\right)}\right)$$

where $\widehat{\mathrm{A}}=A+{I}_{n}$ is the adjacency matrix of the undirected graph $g$ with added self-connections. ${I}_{n}$ is the identity matrix and $\widehat{D}$ is the diagonal node degree matrix of $\widehat{A}$ [25].

## Results and discussion

In this section, we describe a computational test of the performance of the GCN model developed in our study to predict guest accessibility of MOFs using a particular metrics. The proposed method was implemented in Python 3.10 with TensorFlow 2.9.1, Spyder IDE 5.3.1, and some python packages, including Networkx 2.8.4, StellarGraph, REDkit, and RapidMiner 9.19, and Gephi 0.9.

In these tests, we assessed accuracy and loss to evaluate performance. The results facilitated insightful deductions about the potential of MOFGalaxyNet in predicting the guest accessibility of MOFs.

Accuracy is a metric used to evaluate classification models, that is, the fraction of predictions in which the model has succeeded. The accuracy is defined as follows:

12 $$\mathrm{Accuracy}\,=\frac{\mathrm{Number\, of\, correct\, PLD \,predictions}}{\mathrm{Total \,number\, of \,PLD\, prediction}}$$

**Fig. 8:**
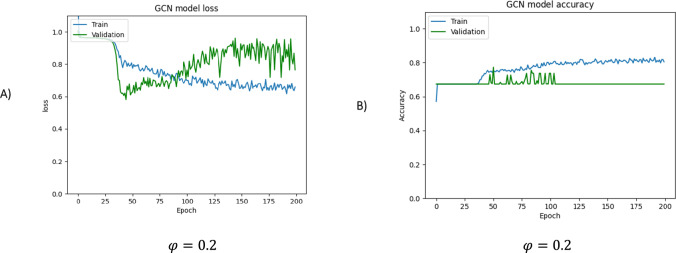
The loss and accuracy curves are presented for the training and validation sets, respectively, for φ = 0.2. **A** The loss function is a measure of the network's performance on the training set and gives an overview of the training process, indicating whether the network is on track. **B** The accuracy curve shows the training and validation accuracies. If there is a considerable difference between the two, it indicates overfitting, and the size of the gap provides an indication of how severe the overfitting is. The overall accuracy percentage achieved is 52.74%

**Fig. 9:**
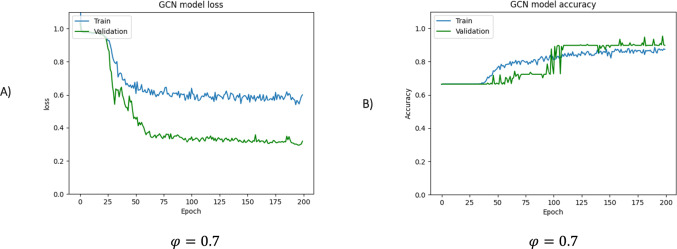
The loss and accuracy curves are presented for the training and validation sets, respectively, for φ = 0.7. **A** The loss function is a measure of the network’s performance on the training set and gives an overview of the training process, indicating whether the network is on track. **B** The accuracy curve shows the training and validation accuracies. If there is a considerable difference between the two, it indicates overfitting, and the size of the gap provides an indication of how severe the overfitting is. The overall accuracy percentage achieved is 86.57%

**Fig. 10:**
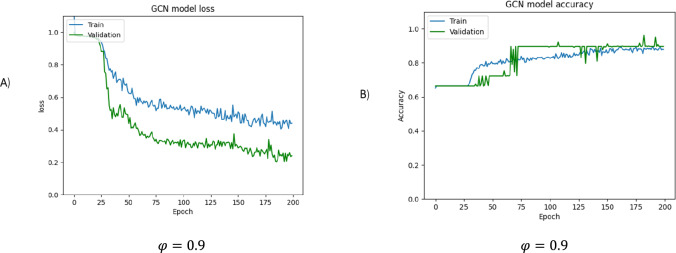
The loss and accuracy curves are presented for the training and validation sets, respectively, for φ = 0.9. **A** The loss function is a measure of the network's performance on the training set and gives an overview of the training process, indicating whether the network is on track. **B** The accuracy curve shows the training and validation accuracies. If there is a considerable difference between the two, it indicates overfitting, and the size of the gap provides an indication of how severe the overfitting is. The overall accuracy percentage achieved is 65.17%

In this study, the GCN model was trained following the proposed approach, and its training progress was monitored using holdout validation with 70% of the data allocated for training. Then its generalization performance was performed over the testing set. To properly utilize the GCN, it is necessary to determine the appropriate values for certain hyperparameters. These hyperparameters include the number of layers, learning rate, training epochs, and batch size. The process of initializing these hyperparameters involves conducting computational tests. These tests involve experimenting with different combinations of values for the hyperparameters and evaluating the performance of the GCN based on specific metrics. By iteratively adjusting and fine-tuning these hyperparameters through computational testing, we can ensure optimal performance of the GCN model. In our experiments, we employed two convolution layers with a size of 32 × 32, a learning rate of 0.01, a dropout probability of 0.5%, 200 training epochs, and a batch size of 16. Additionally, we conducted test runs to showcase the impact of the threshold parameter on the results. Specifically, we performed these test runs using three different threshold values, namely 0.2, 0.7, and 0.9. (Figs. 8, 9, 10).

The loss and accuracy curves are presented to assess the model’s convergence and accuracy. The loss function was calculated during each epoch by evaluating the batches during the forward pass. Inspection of Fig. 8 reveals that with a $\varphi$ value of 0.2, the model performs only poorly on both the training and validation datasets. This indicates underfitting, i.e., the model is too simple and cannot capture the underlying patterns in the data. In this case, the training and validation loss curves are high and do not converge properly.

These curves demonstrate that the social graph contains a high number of edges associated with the value of $\varphi$. However, these edges are not significant and should be removed. Better performance is revealed by the data shown in Fig. 9, which were obtained using a threshold value of 0.7. As shown in the loss plot, the model converges with about 50 epochs, and then, the loss reaches a minimum. The accuracy of the model on training and validation is shown in Fig. 9B. The horizontal axis is the number of iterations, and the vertical axis is the accuracy. A significant deviation between training and validation accuracy is a sign of overfitting, and the degree of this deviation is evidence of the extent of overfitting. In this curve, for the most part, the training curve tracks the validation curve. However, after the about 100 epochs, the validation accuracy becomes more significant than the training accuracy, implying possible small overfitting. In this case, a dropout rate of 0.4 is added to overcome overfitting. As a result, the accuracy of the training set achieved 84.10% and up to 89.55% for the validation set.

Raising the threshold to a higher value, such as 0.9, results in an improvement in performance, as shown in Fig. 10. The figure reveals that when edges with higher weight are retained, nodes have sufficient connections to spread node information effectively, resulting in a higher accuracy than the weight threshold of 0.7.

By comparing the outcomes generated using various weight thresholds, we can deduce that the weight value of 0.9 provides the model with the most favorable direction.

**Fig. 11:**
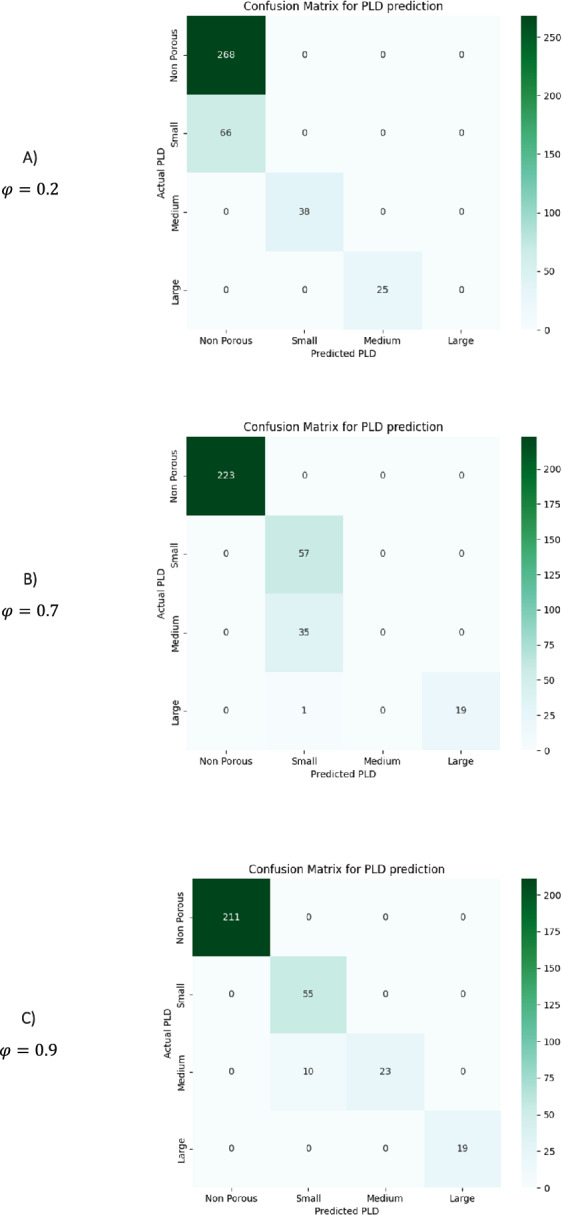
Confusion matrix: The boxes in the matrix diameter show the number of correct predictions by the proposed GCN model. In this training data, 53 MOFs medium PLD MOFs were incorrectly predicted as small PLD

The confusion matrix is a performance evaluation tool used in classification models to assess the model’s accuracy by comparing its predicted output with the actual output [40]. Additionally, the confusion matrix can help identify the errors made by the model, such as misclassifying a certain class, which can help identify and address the root cause of the error. This information can then be used to fine-tune the model and improve its performance on future data. Therefore, we evaluated the confusion matrix using various threshold values in this study. The figures displayed in Fig. 11 depict the confusion matrices that indicate how accurately the GCN model on MOFGalaxyNet detects PLD and the comparatively less accurate MOFs guest accessibility classifications. According to the findings, the matrix performs better than the other matrices when $\varphi =0.9$. Specifically, the matrix correctly predicts all small, large, and non-pores PLDs, but some medium PLDs are identified as small. This validates that using this threshold for both the confusion matrix and accuracy curve yields improved outcomes.

To assess the predictive capabilities of the MOFGalaxyNet GCN model, we compared its performance with several commonly used machine learning models. All the machine learning predictions were conducted using the RapidMiner tools. RapidMiner is a data science platform that provides a range of tools and functionalities for data preparation, machine learning, predictive modeling, and business analytics. The datasets used to evaluate the accuracy of all ML methods and the MOFGalaxyNet are identical, but the preprocessing methods applied to them vary depending on the techniques used.

**Fig. 12:**
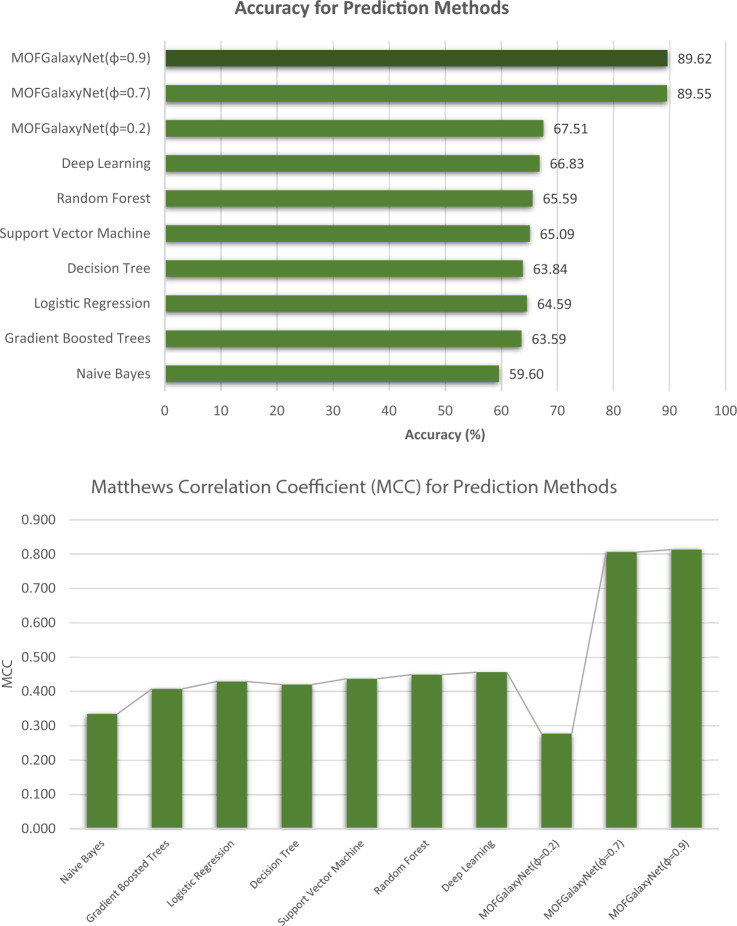
Comparison of MOFGalaxyNet and Other ML Methods by Evaluating Accuracy and Matthews Correlation Coefficient (MCC)

According to the findings, there are notable discrepancies in accuracy between MOFGalaxyNet when $\varphi =0.9$ and other ML techniques, as demonstrated in Table 4 and Fig. 12. To compare the performance of our method, MOFGalaxyNet, with other machine learning methods, we employed the Matthews Correlation Coefficient (MCC) as a robust metric for evaluation. The MCC, which falls within the range of – 1–1, provides valuable insights into the model's ability to handle both class imbalance and binary classification tasks effectively [41]. Our results clearly demonstrate that MOFGalaxyNet outperforms alternative machine learning methods, as evidenced by the higher MCC values achieved. This indicates the model's strong predictive capabilities and suitability for tasks with imbalanced datasets, underscoring its potential for real-world applications where class distributions vary.

**Table 4:** Evaluation of accuracy and Matthews Correlation Coefficient (MCC) using MOFGalaxyNet and compared to other ML algorithms

Model	Accuracy (%) of methods	Matthews correlation coefficient (MCC)
Naive bayes	59.60	0.334
Gradient boosted trees	63.59	0.407
Logistic regression	64.59	0.429
Decision tree	63.84	0.420
Support vector machine	65.09	0.437
Random forest	65.59	0.449
Deep learning	66.83	0.456
MOFGalaxyNet(φ = 0.2)	67.51	0.277
MOFGalaxyNet(φ = 0.7)	89.55	0.806
MOFGalaxyNet(φ = 0.9)	89.62	0.813

**Fig. 13:**
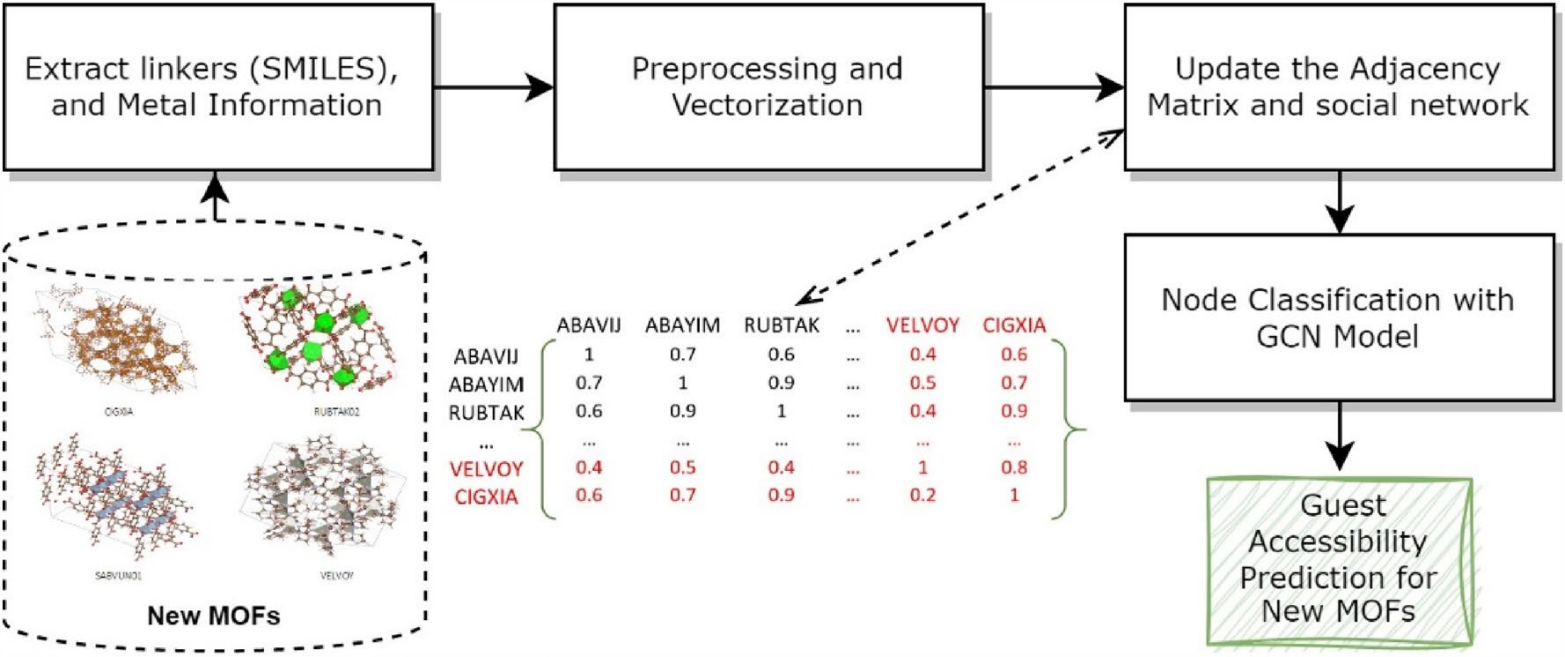
The process of predicting guest accessibility for new MOFs. To predict pore size for MOFs not present in MOFGalaxyNet, the adjacency matrix must be updated by adding the new MOFs to the graph. Relevant MOF information with the same data structure as training data must be extracted and vectorized for similarity measurement prior to updating the matrix. The GCN model is then fed with the updated matrix and new MOFs to make predictions about PLD size

When making predictions for a new MOF, it is important to ensure that the new entry in the data base is represented in the same format as the training data. For example, if the new data has a different structure, it may be necessary to preprocess it to convert it to a graph before making predictions with the GCN. Additionally, it is important to use the same preprocessing steps and hyperparameters that were used during training to ensure that the predictions are consistent and accurate. Figure 13 illustrates the steps for predicting PLD size for new and unknown MOFs. To predict the pore size for MOFs that are not currently present in MOFGalaxyNet, they must be placed into the graph by updating the adjacency matrix. Prior to that, it is necessary to extract relevant MOF information with the same data structure as the training data. Subsequently, this information must be vectorized to prepare it for similarity measurement. Once the adjacency matrix has been updated, the GCN model is fed with the new MOFs and the updated matrix to make predictions regarding PLD size.

**Fig. 14:**
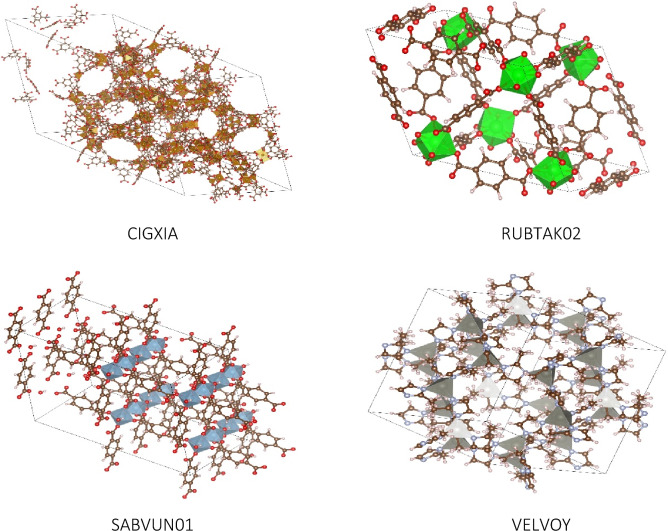
Four MOFs used for model evaluation. The model can predict the PLD size by taking the new matrix and new MOFs information as input. It predicts a “Large” label for both CIGXIA and SABVUM01, and a “Small” label for RUBTAK02 and VELVOY

The performance of the GCN model was evaluated using four MOFs, namely CIGXIA, RUBTAK02, SABVUM01, and VELVOY (Fig. 14). The metal and linker information of these four MOFs were extracted, and the adjacency matrix was updated by adding all relevant similarities between information on these building units and those already present in the matrix. With the new matrix and information on the new building units as model input, the model can predict eventual guest accessibility, which was observed to match the category of the PLD of the corresponding MOFs. To assess this, we computed the porosity of these four MOFs using zeo +  + , which is an open-source software package that is used for computing the geometric properties of porous crystalline systems [42]. Here the PLD was computed using a probe radius of 1.86 Å, which corresponds to the covalent radius of N2. The PLD computed for these systems using zeo +  + are 8.86222 Å and 6.32762 Å for CIGXIA and SABVUM01 respectively which correspond to a “Large” label in the proposed model. Conversely, the PLD of RUBTAK02 and VELVOY are 3.89423 Å and 3.40894 Å, respectively, corresponding to a “Small” label in the proposed model. Evidently, the social network analysis approach that utilizes graph convolutional networks effectively predicts guest accessibility of MOFs in the context of social networking. This method can ultimately be used to accelerate the high throughput screening of MOF materials.

## Conclusion and future direction

In this study, we successfully implemented a new GCN-base approach for predicting guest accessibility of MOFs. We believe that this study will significantly accelerate the high-throughput screening in the development of high-performing MOFs for various host–guest interaction applications. Prior studies have utilized computationally intensive calculations like Grand Canonical Monte-Carlo simulations in high-throughput screening of MOFs for gas separation and permeability [43–48]. We strongly hope that our novel model will compliment these existing approaches in the discovery of high-performing MOFs that can be applied to solve the various societal challenges.

The proposed method comprises two main steps. Firstly, the social network MOFGalaxyNet was built based on MOFs' similarities. Similarities considered graph edges were measured by accounting metal and linker information in a SMILES manner. MOFGalaxyNet can leverage social network analysis to gain better insights into MOF properties. Subsequently, the GCN model was utilized to predict four categories of guest accessibility, namely nonporous, small, medium, and large pores. Eight commonly used machine learning algorithms were selected as baseline models to evaluate the proposed method's performance. The baseline models used for comparison included naive Bayes, generalized linear model, large fast margin, deep learning, decision tree, random forest, gradient boosted trees, and support vector machine. The results showed that the GCN-based method outperformed the other eight models in terms of accuracy. With a predictive accuracy of 86.57%, it can be concluded that the proposed GCN method, based on MOFGalaxyNet, is a robust tool for predicting the guest accessibility of any MOF by predefining the category of the PLD by learning the properties of linkers and metals. The model can predict guest accessibility for MOFs whose linker and metal information are not initially included in the graph. As part of our evaluation process, we have developed a method that offers an improvement over other previously employed ML methods, e.g. the approach proposed in reference [11]. The key advantage of our method is its ability to create a single model instead of relying on three separate binary models, each catering to different categories of PLD. By consolidating the modeling process, we streamline the classification task and enhance efficiency. In the aforementioned work [11], the authors achieved a maximum accuracy of 80.5% using random forest ML methods for their binary classification tasks. Furthermore, this constitutes a significant accomplishment because our method yields superior results by harnessing the enhanced efficiency of a unified model. By encompassing all PLD categories within a single model, we can demonstrate improved accuracy.

Since the proposed approach is a general model, it can accelerate the analysis of MOFs structure and screen MOFs for other design criteria. This work will be further used to design a proxy for predicting other properties of MOFs such as stability prediction (e.g., pressure, temperature, solvent, and water presence), prediction of methane storage, and other criteria.

### Supplementary Information

**Additional file 1: Figure S1.** In this graph representation, nodes correspond to Metal-Organic Frameworks (MOFs), and the connections between them signify interactions within clusters of MOFs. MOFs within the cluster with the highest number of members are highlighted in bold green. This distinct highlighting emphasizes the MOFs central to the largest cluster, potentially indicating their pivotal role within the context of MOF clusters and interactions. **Figure S2.** This figure showcases two distinct clusters of Metal-Organic Frameworks (MOFs) within the network. All MOF nodes are labeled for reference. Nodes highlighted in bold green belong to one cluster, while nodes highlighted in blue belong to the other cluster. This color differentiation emphasizes the existence of two separate clusters and highlights MOFs within each cluster. **Figure S3.** This demonstrates the concept of Tanimoto similarity using Morgan fingerprints with a radius of 2 for two MOFs, namely 'UiO-66' and 'IRMOF-10.' The Tanimoto similarity coefficient between these two MOFs, calculated using their Morgan fingerprints with a radius of 2, is approximately 0.18, indicating a low level of similarity. The Morgan fingerprints, capture the structural features of the molecules, and a higher Tanimoto similarity suggests greater structural similarity between the MOFs. **Table S1.** This table provides comprehensive details for 20 Metal-Organic Frameworks (MOFs), identified by their unique IDs and labeled with their MOF names. **Table S2.** The table highlights network edges, depicting the connections between Metal-Organic Frameworks (MOFs)

## Data Availability

All codes and datasets used in this work are available on a public GitHub repository at https://github.com/MehrdadJalali-KIT/MOFGalaxyNet.
