# Correction: MOFGalaxyNet: a social network analysis for predicting guest accessibility in metal–organic frameworks utilizing graph convolutional networks

**DOI:** 10.1186/s13321-023-00774-0

**Published:** 2023-11-15

**Authors:** Mehrdad Jalali, A. D. Dinga Wonanke, Christof Wöll

**Affiliations:** 1 Institute of Functional Interfaces (IFG), Karlsruhe Institute of Technology (KIT), Eggenstein-Leopoldshafen, Germany; 2 Institute of Nanotechnology (INT), Karlsruhe Institute of Technology (KIT), Eggenstein-Leopoldshafen, Germany

**Correction: Journal of Cheminformatics (2023) 15:94** 10.1186/s13321-023-00764-2

Following publication of the original article [1], we have been informed that Fig. 4 is missing, and instead of Fig. 4, Fig. 3 has been repeated as Fig. 4.

The original article [1] has been corrected.
