# Supplementary material for: MOFGalaxyNet: a social network analysis for predicting guest accessibility in metal–organic frameworks utilizing graph convolutional networks
Source: J Cheminform. 2023 Oct 11;15:94. doi: 10.1186/s13321-023-00764-2 (PMC10568891; doi:10.1186/s13321-023-00764-2)
Supplement: Supplementary file 1 — Additional file 1: Figure S1. In this graph representation, nodes correspond to Metal-Organic Frameworks (MOFs), and the connections between them signify interactions within clusters of MOFs. MOFs within the cluster with the highest number of members are highlighted in bold green. This distinct highlighting emphasizes the MOFs central to the largest cluster, potentially indicating their pivotal role within the context of MOF clusters and interactions. Figure S2. This figure showcases two distinct clusters of Metal-Organic Frameworks (MOFs) within the network. All MOF nodes are labeled for reference. Nodes highlighted in bold green belong to one cluster, while nodes highlighted in blue belong to the other cluster. This color differentiation emphasizes the existence of two separate clusters and highlights MOFs within each cluster. Figure S3. This demonstrates the concept of Tanimoto similarity using Morgan fingerprints with a radius of 2 for two MOFs, namely 'UiO-66' and 'IRMOF-10.' The Tanimoto similarity coefficient between these two MOFs, calculated using their Morgan fingerprints with a radius of 2, is approximately 0.18, indicating a low level of similarity. The Morgan fingerprints, capture the structural features of the molecules, and a higher Tanimoto similarity suggests greater structural similarity between the MOFs. Table S1. This table provides comprehensive details for 20 Metal-Organic Frameworks (MOFs), identified by their unique IDs and labeled with their MOF names. Table S2. The table highlights network edges, depicting the connections between Metal-Organic Frameworks (MOFs) [file 13321_2023_764_MOESM1_ESM.pdf]

Additional information

1. Additional figures

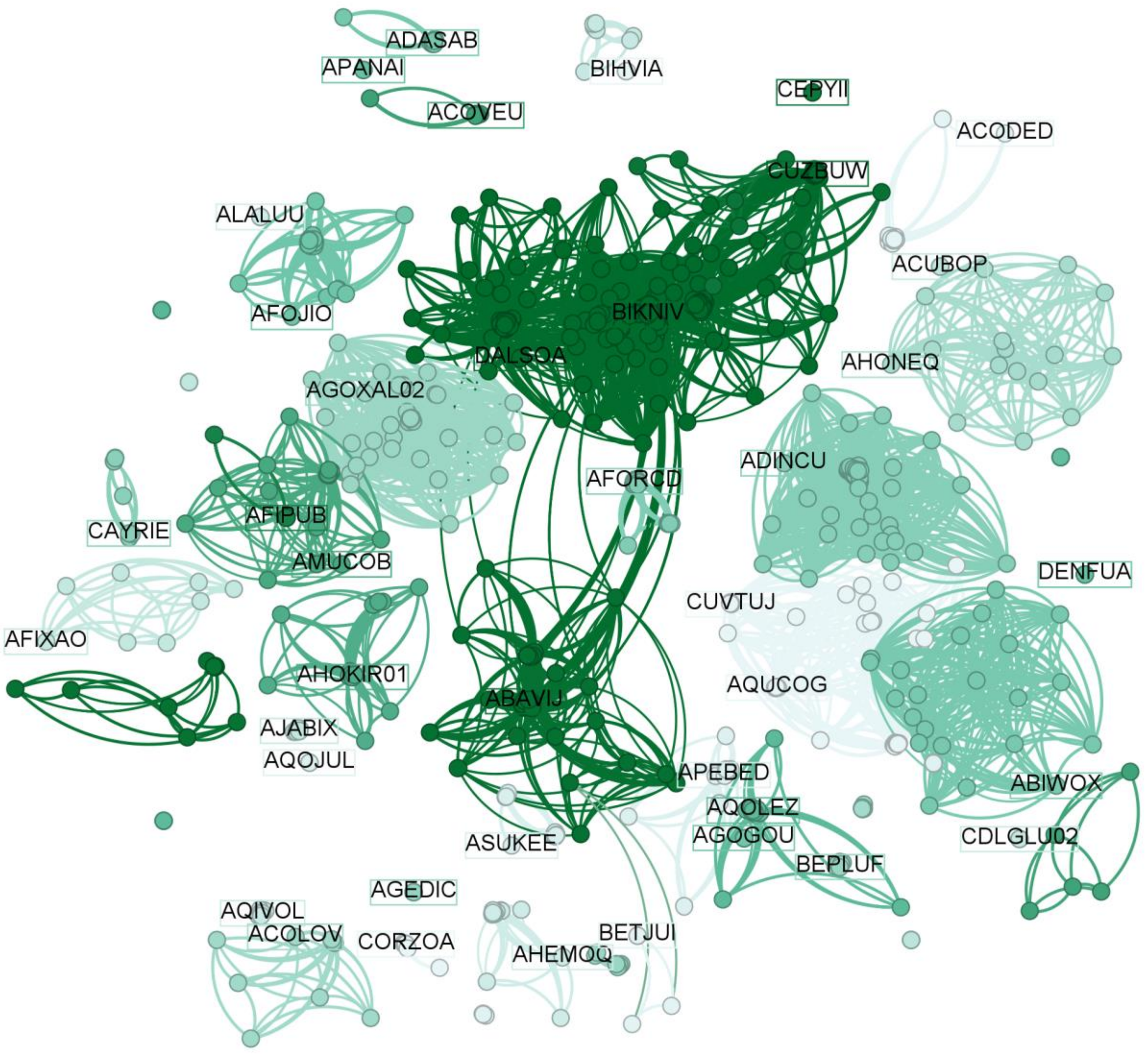

Figure S1. In this graph representation, nodes correspond to Metal-Organic Frameworks (MOFs), and the connections between them signify interactions within clusters of MOFs. MOFs within the cluster with the highest number of members are highlighted in bold green. This distinct highlighting emphasizes the MOFs

central to the largest cluster, potentially indicating their pivotal role within the context of MOF clusters and interactions.

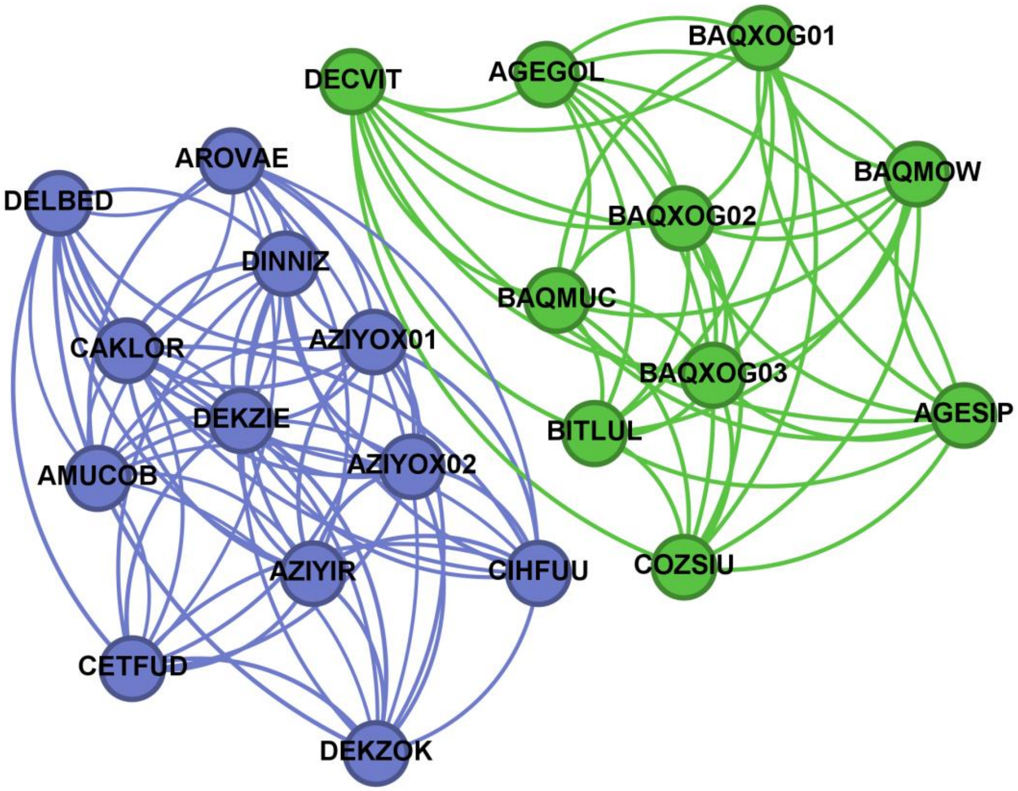

Figure S2 This figure showcases two distinct clusters of Metal-Organic Frameworks (MOFs) within the network. All MOF nodes are labeled for reference. Nodes highlighted in bold green belong to one cluster, while nodes highlighted in blue belong to the other cluster. This color differentiation emphasizes the existence of two separate clusters and highlights MOFs within each cluster.

MOF Name	Linker SMILE	Morgan Fingerprint
UiO-66	**CC(C)(C)Nc1ccc(C(=O)O)cc1.c1ccc(cc1)C(=O)O.Cc1cccc(c1)C(=O)O**	[illegible]
IRMOF-10	**O=C(C)Oc1ccc2c(c1)nc(cc2)C(=O)O**	[illegible]

$$\boldsymbol{Tanimoto\ Similarity\ (UiO-66, IRMOF-10) = \frac{(Number\ of\ Common\ Bits)}{(Total\ Number\ of\ Unique\ Bits\ in\ Both)}}$$

Figure S3. This demonstrates the concept of Tanimoto similarity using Morgan fingerprints with a radius of 2 for two MOFs, namely 'UiO-66' and 'IRMOF-10.' The Tanimoto similarity coefficient between these two MOFs, calculated using their Morgan fingerprints with a radius of 2, is approximately 0.18, indicating a low level of similarity. The Morgan fingerprints, capture the structural features of the molecules, and a higher Tanimoto similarity suggests greater structural similarity between the MOFs.

2. Additional tables

Table S1. This table provides comprehensive details for 20 Metal-Organic Frameworks (MOFs), identified by their unique IDs and labeled with their MOF names. The columns in the table represent the following network parameters:

Id: The unique identifier for each MOF.
Label: The label corresponds to the MOF name.
Degree: Indicates the degree of each MOF, representing the number of connections within the network.
Modularity Class: Specifies the modularity class to which each MOF belongs, aiding in network community identification.
Eccentricity: Reflects the eccentricity of each MOF node in the network, describing its position relative to other nodes.
Closeness Centrality: Measures how close each MOF is to other MOFs in the network.
Harmonic Closeness Centrality: Quantifies the harmonic closeness centrality of each MOF, indicating its centrality within the network.
Betweenness Centrality: Measures the betweenness centrality of each MOF, highlighting its role in connecting different parts of the network.
Cluster-ID: Identifies the cluster or group to which each MOF belongs within the network.
Weighted Degree: This represents the weighted degree of each MOF, considering the strength of connections and their number.

Id	Label	Degree	modularity_class	Eccentricity	closeness centrality	Harmonicclosness centrality	betweenness centrality	Cluter-ID	Weighted Degree
**0**	ABAVIJ	34	0	10	0.25405	0.347032	0	218	32.23106
**1**	ABAVOP	34	0	10	0.25405	0.347032	0	218	32.23106
**2**	ABAVUV	34	0	10	0.25405	0.347032	0	218	32.23106
**3**	ABAYIM	5	1	1	1	1	0	96	4.930149
**4**	ABAYIO	52	111	8	0.270801	0.377133	89.19423	224	47.81134
**5**	ABAYOS	5	1	1	1	1	0	96	4.930149
**6**	ABAYOS01	5	1	1	1	1	0	96	4.930149
**7**	ABAYOU	54	111	8	0.278002	0.385248	2605.994	224	49.12404
**8**	ABEFUL	2	2	1	1	1	0	156	1.999686
**9**	ABEMIF	53	111	8	0.271226	0.379065	201.9442	224	48.38397
**10**	ABIWOX	21	3	1	1	1	1	97	20.01179

**11**	ABUFOU	4	4	1	1	1	0.333333	19	3.418521
**12**	ABUFUA	4	4	1	1	1	0.333333	19	3.416963
**13**	ABUWOJ	70	5	9	0.325472	0.478693	790.9795	223	63.2869
**14**	ACAFAO	2	6	1	1	1	0	183	1.55
**16**	ACAJOG	15	3	2	0.777778	0.857143	0	97	10.86703
**17**	ACAKEX	15	3	2	0.777778	0.857143	0	97	10.86703
**18**	ACAKUM	6	7	1	1	1	0	39	5.449746
**19**	ACALAT	6	7	1	1	1	0	39	5.455553

Table S2. The table highlights network edges, depicting the connections between Metal-Organic Frameworks (MOFs). Each edge consists of a source MOF, a target MOF, and a weight, quantifying the similarity between the connected MOFs. These edge details provide valuable insights into the network's relationships and similarities among MOFs.

Source	Target	Weight
**1548**	1549	1
**1392**	1548	0.8875
**1283**	1548	0.8875
**391**	1548	1
**367**	475	0.996773
**368**	475	0.996773
**1884**	1886	1
**1884**	1885	0.998924
**1885**	1886	0.998924
**367**	368	1
**391**	1549	1
**391**	1392	0.8875
**391**	1283	0.8875

**1392**	1549	0.8875
**1283**	1549	0.8875
**1283**	1392	1
**959**	1537	0.933458
**959**	1439	0.949799
**959**	1438	0.949799
**959**	1328	0.929283
**959**	1327	0.938387
**959**	1326	0.938387
**959**	1205	0.978752
**803**	959	0.982194
**1328**	1537	0.981106
**1328**	1439	0.981655
**1328**	1438	0.981655
**1327**	1328	0.997023
**1205**	1328	0.962143
**803**	1328	0.962103
**1326**	1328	0.997023
**1438**	1537	0.998239
**1438**	1439	1
**1327**	1438	0.993054
**1205**	1438	0.991287
**803**	1438	0.989087
**1326**	1438	0.993054
**1326**	1537	0.991969
**1326**	1439	0.993054
**1326**	1327	1
**1205**	1326	0.976966
**803**	1326	0.975498
**1439**	1537	0.998239
